# Production of Mature Recombinant Human Activin A in Transgenic Rice Cell Suspension Culture

**DOI:** 10.3390/cimb46020074

**Published:** 2024-01-30

**Authors:** Van Giap Do, Moon-Sik Yang

**Affiliations:** 1 Apple Research Institute, National Institute of Horticultural and Herbal Science, Rural Development Administration, Daegu 39000, Republic of Korea; giapbio@korea.kr; 2 Department of Bioactive Material Science, Jeonbuk National University, Jeonju 54896, Republic of Korea

**Keywords:** human activin A, rice a-amylase 3D promoter, transgenic rice cell, suspension culture

## Abstract

Activin A belongs to the transforming growth factor (TGF) family member, which exhibits a wide range of biological activities, including the regulation of cellular proliferation and differentiation and the promotion of neuronal survival. The isolation of AA from natural sources can only produce limited quantities of this bioactive protein. In this study, the whole gene of the precursor form of recombinant human activin A (rhAA) contains a signal peptide, and a pro-region and a mature region were cloned into an expression vector under the control of the rice α-amylase 3D (RAmy3D) promoter. To obtain the mature (active) form of rhAA, an enterokinase cleavage site was inserted between the pro-region and mature region of rhAA. The rice seed (*Oryza sativa* L. cv. Dongjin) was transformed with recombinant vectors by the Agrobacterium-mediated method, and the integration of the target gene into the plant genome was confirmed by genomic PCR. The transcript expression of rhAA in transgenic rice calli was confirmed by a Northern blot analysis of mRNA. The production of rhAA was verified by Western blot analysis and ELISA. The accumulation of secreted rhAA in the culture medium was purified by Ni^2+^—NTA. The mature form of AA was released from the precursor form of rhAA after proteolytically processing with enterokinase. Western blot shows that the mature AA was split into monomer and homodimer with molecular weights of 14 kDa and 28 kDa under reducing and non-reducing conditions, respectively. These results suggest that the mature form of rhAA could be produced and purified using transgenic rice cell suspension culture.

## 1. Introduction

Activins, belonging to the TGFβ family, are formed by homodimers or heterodimers of various β subunit isoforms. The β-subunit precursor comprises a 290 amino acid pro-region and a 116 amino acid C-terminus (mature-region) [1]. Activin A (AA) is a homo-dimeric protein comprising a βA subunit of inhibin in the pro-region that is linked by basic cleavage sites to the mature region. Mature AA consists of two residue βA subunits (βA-βA). Like other members of the TGF-β superfamily, activins exist as dimeric proteins, comprising two βA subunits (activin A), two βB subunits (activin B), or a combination of a βA and a βB subunit (activin AB). In addition, a βC, a βD, and a βE subunit have been identified, although the corresponding proteins have not been characterized yet. The βA and βB subunits can also form dimers with a homologous α subunit, resulting in the formation of inhibin A (αβA) or inhibin B (αβB) [2].

Activin A demonstrates diverse biological activities, contributing to mesoderm induction, neural cell differentiation, bone remodeling, hematopoiesis, and reproductive physiology. They play a pivotal role in producing and regulating hormones such as FSH, LH, GnRH, and ACTH. In normal cellular function, activin A signaling is tightly regulated to uphold cellular and tissue health while suppressing tumor growth. The disruption of activin A signaling has been linked to tumor formation and progression, making activin A availability a crucial target for diagnostic and therapeutic development [3]. Cells expressing activin A include fibroblasts, endothelial cells, hepatocytes, vascular smooth muscle cells, macrophages, keratinocytes, osteoclasts, bone marrow monocytes, prostatic epithelium, neurons, chondrocytes, osteoblasts, Leydig cells, Sertoli cells, and ovarian granulosa cells [4,5,6,7]. Similar to other members of the superfamily, activins interact with two types of cell surface transmembrane receptors (types I and II), each possessing intrinsic serine/threonine kinase activities in their cytoplasmic domains. Activin type I receptors include ACVR1, ACVR1B, and ACVR1C, while activin type II receptors comprise ACVR2A and ACVR2B. The biological activity of activin A can be counteracted by inhibins and the diffusible TGF-β antagonist, Follistatin [8].

The use of plant cells for producing naturally occurring or recombinant compounds and proteins has garnered increased attention over the past decades. Plant cell-suspension culture stands out as an alternative platform to mammalian cells for biopharmaceutical production. This system ensures sterile in vitro conditions with high-level containment and offers cost-effective and simplified downstream processing and purification [9]. Utilizing suspension-cultured cells as an expression system holds the potential to minimize protein and N-glycan heterogeneity due to the uniformity in cell size and type [10,11]. Additionally, this approach is rapid, eliminating the need to regenerate and characterize transgenic plants, allowing for the generation of productive cell lines within a few months [12].

Activin A has been isolated from various sources, including porcine follicular [13] and ovine amniotic fluid [14]. Despite its current availability in the commercial market in limited quantities, the protein comes at a high cost and is only offered in amounts that may not be feasible for many research applications. This limitation restricts its potential development in diagnostic or therapeutic contexts. This study aimed to address these drawbacks by producing recombinant human activin A (rhAA) in substantial quantities using transgenic rice cell-suspension culture. The recombinant protein was efficiently secreted into the culture medium, allowing for straightforward purification. Consequently, this approach presents a promising strategy for the cost-effective large-scale production of biopharmaceutical rhAA, overcoming the challenges associated with the current commercial availability of the protein. 

## 2. Materials and Methods

### 2.1. Vector Construction

The sequence of the rhAA gene was synthesized by Bioneer Co., Ltd. (Daejeon, Republic of Korea). The whole gene of the precursor form of recombinant human activin A (rhAA) contains a pro-region, and the mature region was cloned into an expression vector under the control of the rice α-amylase 3D (RAmy3D) promoter. To obtain the mature form of rhAA, the enterokinase cleavage site (Asp-Asp-Asp-Asp-Lys, termed as D4K) was inserted between the pro-region and mature region of rhAA. Overlap PCR was conducted to amplify the whole gene of the precursor form of rhAA using designed specific primer sets (Table 1). To facilitate the cloning, restriction enzyme sites BsaI, XbaI to 5′-end of forward primer complementary with DNA sequence on the signal peptide of pro-region, and SacI, BsaI, KpnI to 3′-end reverse primer complementary with DNA sequence on the mature region of rhAA were added. In addition, for recombinant protein purification, 6xHis-tag was added at the C-terminal of the gene. PCR products were cloned into a pGEM-T easy vector (Promega, Madison, WI, USA), and the sequence gene was confirmed by DNA sequence analysis (Genotech, Seoul, Republic of Korea). For the construction of recombinant plant expression vectors, the digested fragments of the cloned gene with XbaI and KpnI were introduced into the same sites of the plant expression vector under the control of the RAmy3D promoter, with 3′UTR of the RAmy3D gene as the terminator. The resulting recombinant plant expression vectors were generated, and pYMD317 and pYMD318 (containing 6xHis-tag) were introduced into *Agrobacterium tumefaciens* LBA4404 by triparental mating method [15].

### 2.2. Rice Calli Transformation

Rice calli (*Oryza sativa* L. cv. Dongin) were prepared and transformed by Agrobacte-rium-mediated transformation as previously described [16]. Briefly, rice calli were induced in an N6CI medium containing N6 salts and vitamins (Duchefa, Haarlem, The Netherlands) supplemented with sucrose (30 g/L), kinetin (0.02 mg/L), 2,4-D (2 mg/L), and phytagel (2.3 g/L) at pH 5.7 for 2 weeks. The rice embryogenic calli were detached and transferred to a fresh N6CI medium for 1 week before the period to Agrobacterium-mediated transformation. The Agrobacterium strain LBA4404 harboring the rice expression vectors was inoculated in Luria Bertani (LB) broth medium containing kanamycin (50 mg/L) and rifampicin (50 mg/L) and incubated at 28 °C overnight. 

The Agrobacterium cells were collected by centrifuge and then suspended in an N6CI liquid medium containing 200 µM of acetosyringone. The rice calli were soaked into the suspension of Agrobacterial cells for 10 min, gently shaking every 2 min interval for Agrobacterial cell infection. After infection, the suspended medium containing Agrobacterium was removed, and the rice calli were blotted on sterile Whatman filter paper for drying. The infected rice calli were transferred onto a co-culture medium (N6CO) containing N6 salts and vitamins supplemented with sucrose (30 g/L), casamino acids (1 g/L), kinetin (0.02 mg/L), 2,4-D (2 mg/L), glucose (10 g/L), phytagel (2.3 g/L), and acetosyringone (100 µM) and were incubated in the dark at 25 °C for 3–5 days. After co-cultivation, the calli were washed three times with triple distilled water and then one time with sterilized water containing cefotaxime (500 mg/L). The infected rice calli were blotted on sterile filter Whatman paper and transferred on the selection medium (N6SE) containing hygromycin B (50 mg/L) and cefotaxime (250 mg/L) for transgenic selection. The appearance of small transgenic hygromycin-resistant calli commenced after 3–4 weeks on the selection medium.

### 2.3. Polymerase Chain Reaction (PCR) Analysis

Genomic DNA was isolated from wild-type and putative transgenic rice calli using the ZR Genomic DNA Kit (Zymo Research Corp, Irvine, CA, USA). The quality of genomic DNA was checked on 1.0% agarose gel before doing genomic DNA PCR. The presence of rhAA was determined using gene-specific forward and reverse primers, which were used for the construction of the recombinant plant expression vectors. PCR was carried out in a 20 μL volume containing 100 ng of genomic DNA, 10 pmol of the primer sets specific for the target genes, and 10 μL of 2× GoTaq master mix containing dNTPs and polymerase. PCR was processed as follows: 95 °C for 5 min, followed by 30 cycles of (95 °C for 1 min, 58 °C for 1 min 30 s, and 72 °C for 1 min), and final extension at 72 °C for 2 min. PCR products were analyzed by 1.0% agarose gel electrophoresis and visualized by ethidium bromide staining under ultraviolet light using a Gel Doc^TM^ EZ Imager (Bio-Rad Laboratories, Inc., Hercules, CA, USA).

### 2.4. Establishment and Induction of Rice Cell Suspension Culture

For the putative transgenic lines after confirmation using genomic DNA PCR, transgenic rice calli lines harboring the target genes (rhAA) were cultured on a rotary shaking incubator at 110 rpm at 28 °C in darkness. To propagate the individual cell line, the rice cell suspension was cultured in a 50 mL volume of an N6 medium using 300 mL flasks as previously described [17]. To maintain propagation, the suspension cell line was subcultured every 7 days by transferring 10 mL inoculums to a fresh medium. For the induction of gene expression, the rice calli were removed from the cell suspension by aspiration, and the rice cells were transferred to a fresh N6 (–S) medium (sucrose starvation). After 7 days, the culture supernatant from the culture medium of induced rice cells under sugar starvation was filtrated through the multiple layers of Whatman filter paper. The secreted proteins onto the culture medium were collected by centrifuge at a high speed of 13,200 rpm for 15 min at 4 °C. The supernatant was carefully sampled to prevent contamination of the debris.

### 2.5. Northern Blot Analysis

Northern blotting analysis was conducted to determine the transcription of target genes in transgenic rice calli. To induce mRNA transcription, transgenic rice calli containing target genes under the regulation of a Ramy3D promoter were induced with sugar starvation for 7 days. Total RNA was extracted from the calli of wild-type and transgenic rice using TRIzol^®^ Reagent (Life Technologies, Van Allen Way, CA, USA). A total of 30 µg of RNA samples were separated by electrophoresis on 1.2% formaldehyde-agarose gel and then transferred to a Hybond N^+^ membrane (Amersham Pharmacia Biotech, Piscataway, NJ, USA). The membrane was hybridized overnight with target probes labeled with random-primed ^32^P-dCTP (Promega) at 65 °C in a hybridization buffer (pH 7.4) containing 1 mM EDTA, 250 mM Na_2_HPO_4_·7H_2_O, 1% hydrolyzed casein, and 7% SDS. The membrane was washed twice with the following washing buffers: washing buffer A (2× SSC, 0.1% SDS), washing buffer B (2× SSC, 1% SDS), and washing buffer C (0.1× SSC, 0.1% SDS) at 65 °C, with 15 min for each washing buffer. The membrane was then exposed to X-ray film (Eastman Kodak, Rochester, NY, USA), and the hybridized bands of mRNA were visualized via autoradiography.

### 2.6. SDS–PAGE and Western Blot Analysis

For the expression analysis of recombinant protein, an aliquot of 20 µL of secreted protein from induced rice suspension (supernatant culture media) or 15 µg of total soluble protein (TSP) extracts from induced rice calli used SDS-PAGE and Western blot analysis. The SDS-PAGE and Western blot analyses were processed as previously described [18] with slight modifications. The TSP, along with pre-stained molecular weight markers, was loaded and separated via 10% sodium dodecyl sulfate-polyacrylamide gel electrophoresis (SDS-PAGE) at 120V for 2–3 h on a Tris-glycine buffer (25 mM Tris-Cl, 250 mM glycine and 0.1% SDS, pH 8.3). For the detection of the monomeric form of proteins, these proteins were mixed with a reducing buffer and boiled for 10 min before performing gel electrophoresis.

The separated protein bands were electroblotted onto a Hybond™ C nitrocellulose membrane (Amersham Pharmacia Biotech, Piscataway, NJ, USA) using Trans-Blot^®^ SD Semi-Dry Transfer Cell (Bio-Rad, Hercules, CA, USA) at 15V for 30 min or using a mini-transblot apparatus (Bio-Rad, Hercules, CA, USA) at 150 mA for 2–3 h in a transfer buffer (50 mM Tris, 40 mM glycine, and 20% methanol). The protein-blotted membranes were blocked with 5% non-fat dry milk in a TBST buffer (20 mM Tris-Cl, pH 7.5, 500 mM NaCl, 0.05% Tween20) at room temperature for overnight incubation. The membranes were washed three times with the TBST buffer and subsequently incubated with a primary specific antibody (Ab) (inhibin beta-A Ab or human AA precursor Ab) diluted 1:5000 in a TBST buffer containing 5% non-fat dry milk at room temperature for 2 h. After washing three times with the TBST buffer, the membranes were finally incubated with a secondary antibody conjugated to alkaline phosphate diluted 1:7000 in the TBST buffer containing 5% non-fat dry milk at room temperature for 2 h. The membranes were colorimetrically detected using a premixed BCIP/NBT color development solution (B6404, Sigma-Aldrich, St. Louis, MO, USA).

### 2.7. Quantification of Recombinant Human Activin A

To evaluate rhAA presence in the culture medium, an indirect ELISA was performed. Microtiter plates (NUNC, Netherlands) were coated with 100 µL per well of 0.2 µg standard recombinant human activin A (Isokine™, ORF Genetics, Kópavogur, Iceland), along with rhAA produced from transgenic rice cells in a coating buffer (15 mM Na_2_CO_3_, 35 mM NaHCO_3_, pH 9.6), and were placed at 4 °C overnight. On the following day, the wells were washed three times with PBST (PBS buffer with 0.05% Tween 20). The plate was then given 200 µL blocking buffer containing 0.1% bovine serum albumin and left at room temperature for 2 h and then washed 3 times with PBST. Subsequently, 100 µL per well of a 1:1000 dilution of inhibin β-A monoclonal antibody (Santa Cruz Biotechnology, Inc., Dallas, TX, USA) was added. After incubating at room temperature for 2 h, the plate was washed with PBST, and 100 µL of a 1:7000 dilution of alkaline phosphatase-conjugated goat anti-mouse IgG (W4021, Promega, Madison, WI, USA) was added to wells. The microplate was incubated at room temperature for 2 h and washed three times with PBST. The color was developed by the addition of 100 µL per well of phosphatase substrates (S0942, Sigma-Aldrich, St. Louis, MO, USA). Optical density was measured at a wavelength of 405 nm using an ELISA reader (Sunrise, Tecan, Männedorf, Switzerland).

### 2.8. Purification of Recombinant Human Activin A

rhAA was purified as histidine-tagged (His-tag) fusion protein from rice cell culture medium using affinity chromatography on a Ni^2+^—NTA agarose (Qiagen, Hilden, Germany) column, following the manufacturer’s instructions. Briefly, 50 mL of a 7-day induced culture medium was centrifuged at 13,200 rpm at 4 °C for 20 min and filtrated through a 0.2 µm filter to remove debris. The column was washed out of the preservative, and 700 µL of Ni^2+^—NTA agarose resin was added into the open column. After equilibrating with 5 mL of 10 mM Imidazole, the prepared culture medium (added 1M Imidazole to the culture medium to obtain the final 10 mM) was loaded to the purification column at a flow rate of about 0.5 mL/min. The Ni^2+^—NTA agarose column was washed with 10 mL of 10 mM Imidazole. Binding proteins were eluted using 250 mM Imidazole in a total volume of 1 mL. Fractions were collected, and purity was visually assessed by SDS-PAGE and Western blot analysis.

### 2.9. Enterokinase Treatment of rhAA in Transgenic Rice Calli 

The secreted rhAA in the suspension culture medium was concentrated using Amicon^®^ Ultra centrifugal filter 50 K (Merck Millipore, Burlington, MA, USA). The enterokinase treatment was performed with a total volume of 178 µL of the concentrated sample, 20 µL of a 10× digestion buffer, and 2µL of recombinant enterokinase at 23 °C for 12 h. A control protein, tumor necrosis factor-alpha (TNFα), was fused to enhanced green fluorescent protein by a cleavage site enterokinase (termed as EGPF-D4K-TNFα) for conducting control digest in parallel with the experimental sample.

## 3. Results

### 3.1. Construction of Rice Expression Vectors for rhAA

**Figure 1:**
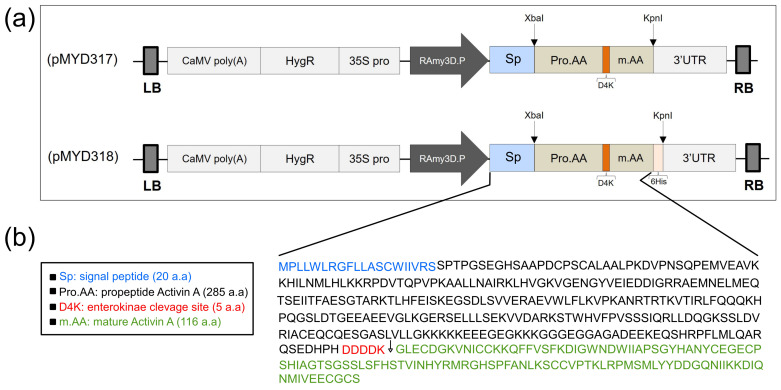
(**a**) Schematic representation of the constructs pMYD317 and pMYD318 within the T-DNA region harboring the target gene rhAA and selection marker gene (HygR) under the control of the rice α-amylase 3D (RAmy3D) promoter and the cauliflower mosaic virus 35S (35S pro) promoter, respectively. LB, T-DNA left border; 35S polyA, the terminator of the 35S gene; HygR, hygromycin phosphotransferase; 35S pro, cauliflower mosaic viral (CaMV) 35S promoter; 3′-UTR, 3′-untranslated region of the rice α-amylase 3D gene; RB, T-DNA right border. (**b**) The precursor rhAA contains a signal peptide, pro-region, and mature region located between the rice RAmy3D promoter and 3′UTR terminator.

**Figure 2:**
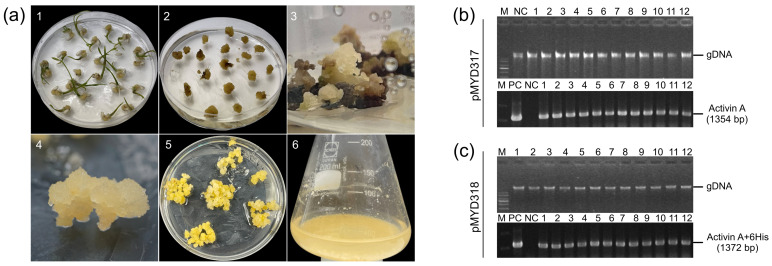
(**a**) The stepwise establishment of transgenic rice calli for the production of rhAA using cell suspension culture. The transgenic rice calli were established using Agrobacterium-mediated transformation. (**1**) Callus induction of rice seeds on the callus induction medium N6CI; (**2**) embryonic calli were detached from germinated seeds using for transformation via Agrobacterium-mediated transformation; (**3**) the appearance of resistance calli on the selection medium contained hygromycin B; (**4**,**5**) the propagation of transgenic rice calli on the selection medium; (**6**) the establishment and induction of rice cell suspension culture. (**b**,**c**) The selection of rice transgenic calli lines using genomic DNA PCR to detect the target gene (hAA) from the extracted genomic DNA of transformed lines. Lane M, 1 Kb plus DNA ladder marker; lane PC, plasmid DNA of pMYD317 and pMYD318 vectors as a positive control; lane NC, genomic DNA of non-transgenic callus (wild-type) used as a negative control; lanes 1–12, independent rice transgenic calli lines.

The gene encoding rhAA protein after cloning into the pGEM T-easy vector (Promega, Madison, WI, USA) and confirmed by DNA sequencing was cloned into the rice expression vector driven by the RAmy3D promoter, resulting in pMYD317 and pMYD318 (Figure 1a). For purification purposes, the plant expression vector pMYD318, containing a sequence of six histidine tags (6xHis), was added at the 3′-end of the rhAA gene. The precursor form of rhAA contains a signal peptide, pro-region, and mature region (Figure 1b) and was under-regulated by the rice RAmy3D promoter, with 3′UTR of the RAmy3D gene as the terminator. The hygromycin phosphotransferase gene (HygR) provides hygromycin B resistance in plants as a selection marker. The binary plant expression vectors pMYD317 and pMYD318 were transformed into *Agrobacterium tumefaciens* LBA4404 using pRK2013 as mobilization helper by the tri-parental mating method. These binary plant expression vectors were introduced into rice calli via the Agrobacterium-mediated method. Putative transgenic calli appeared 2–3 weeks on the selection medium containing hygromycin B (Figure 2a).

### 3.2. Genomic PCR Analysis of Putative Transgenic Rice Calli of rhAA

PCR analysis was performed to confirm the presence of the rhAA gene in the genomic DNA of putative transgenic rice calli lines. All putative transgenic rice calli lines (12 lines of each construction) showed the presence of 1345 bp-band (pMYD317) (Figure 2b) and 1372 bp-band (pMYD318) (Figure 2c). This suggests that the rhAA gene was integrated into the chromosomal DNA of the transgenic plants. No bands were detected in the genomic DNA of untransformed rice calli (NC), which were used as a negative control. Seven rice callus lines per construct were selected for further analysis and established as suspension cells. 

### 3.3. Northern Blot Analysis of rhAA in Transgenic Rice Calli

**Figure 3:**
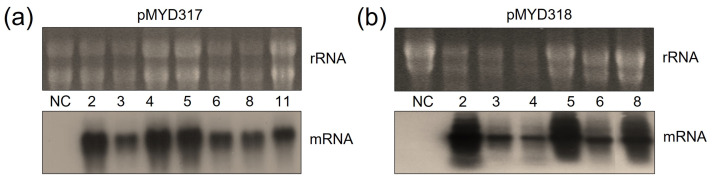
Northern blot analysis to evaluate the expression of rhAA mRNA in transgenic rice cell lines of pMYD317 (**a**) and pMYD318 (**b**). Lane NC shows total RNA extracted from non-transformed rice cells (negative control); other lanes contain RNA extracts of transgenic rice cells.

The mRNA transcript of the rhAA gene was identified by Northern blot analysis. To evaluate the transcript expression of the rhAA gene, seven lines of pMYD317 and six lines of pYMD318 were induced under sugar starvation conditions for 7 days. Total RNA was extracted from the calli of wild-type and transgenic rice calli. The ribosomal RNA (rRNA) was used to indicate an equal amount of total mRNA among samples that were subjected to RNA blot analysis. Transcript signals were detected in all transgenic cell lines, and no signal of mRNA was detected in the wild-type cell line (NC) (Figure 3). The mRNA expression of AA was variable between lines. Its expression level was strong in lines 2, 4, 5, and 11 of the pMYD317 construct (Figure 3a) and in lines 2, 5, and 8 of the pMYD318 construct (Figure 3b).

### 3.4. Production of rhAA in Transgenic Rice Cell Suspension Culture

**Figure 4:**
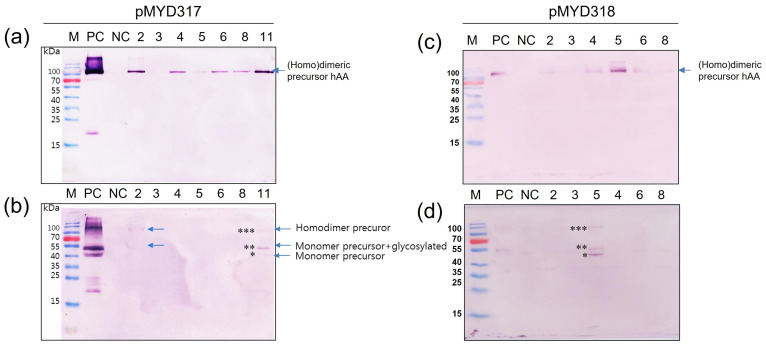
Screening of transgenic rice cell lines has a high expression of rhAA. Seven days of the induced supernatant of cell lines were collected for Western blot analysis. Western blot analyses were conducted using AA precursor Ab as the primary Ab to detect rhAA by binding to the precursor form of AA. The expression of rhAA was detected under non-reducing conditions (**a**,**c**) and reducing conditions (**b**,**d**) in both pMYD317 and pMYD318. Lane M, a pre-stained protein marker; Lane PC, 100 ng of recombinant human AA; NC, culture medium from the non-transgenic cell line; other lanes, transgenic cell lines. Single, double, and triple asterisks in (**b**,**d**) indicate the monomer precursor, glycosylated forms of the monomer precursor, and the homodimer precursor of rhAA, respectively.

To determine whether rhAA was synthesized and secreted into the culture medium in a sucrose-dependent manner, Western blot analysis was assessed 7 days after the induction of transgenic cell suspension (Figure 4). Under the non-reducing conditions, the homodimeric precursor of rhAA was detected with a molecular weight of approximately 90 kDa (Figure 4a,c). Under reducing conditions, a monomer of precursor rhAA was detected with a molecular weight of approximately 45 kDa (Figure 4b,d). No bands were detected from untransformed rice calli as a negative control. The highest expression was observed in cell line 11 of pMYD317 and cell line 5 of pMYD318. 

**Figure 5:**
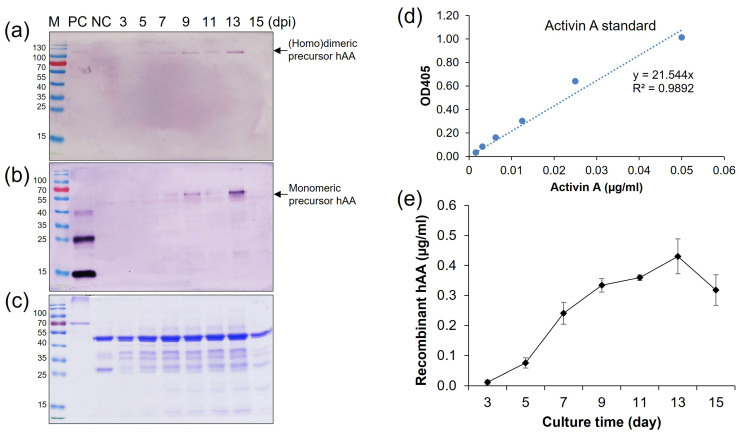
Time-course production of rhAA in the transgenic rice cell suspension culture medium. The secreted rhAA in the suspension culture medium was quantified via Western blot (**a**,**b**), SDS-PAGE (**c**), and ELISA (**d**,**e**) over a period of 15 days under sugar starvation.

To estimate the maximum production phase by time-course induction, the high expressed cell line (elite line) number 5 of pMYD318 was induced in the N6 (–S) medium, and the supernatant was collected on two-day intervals from 3 days post-induction (dpi) to 15 dpi. Quantification of protein expression was analyzed by SDS-PAGE, Western blot, and ELISA analysis (Figure 5a–e). The expression level of rhAA steadily increased in correlation with induction time and reached its highest at 13 dpi and then quickly dropped thereafter (Figure 5). The amount of secreted rhAA into the culture medium was measured by ELISA, along with a standard recombinant human activin A (Isokine™, ORF Genetics) (Figure 5d). The highest level was 0.43 µg/mL at 13 dpi (Figure 5e).

### 3.5. Protein Purification by Affinity Chromatography Ni^2+^—NTA

**Figure 6:**
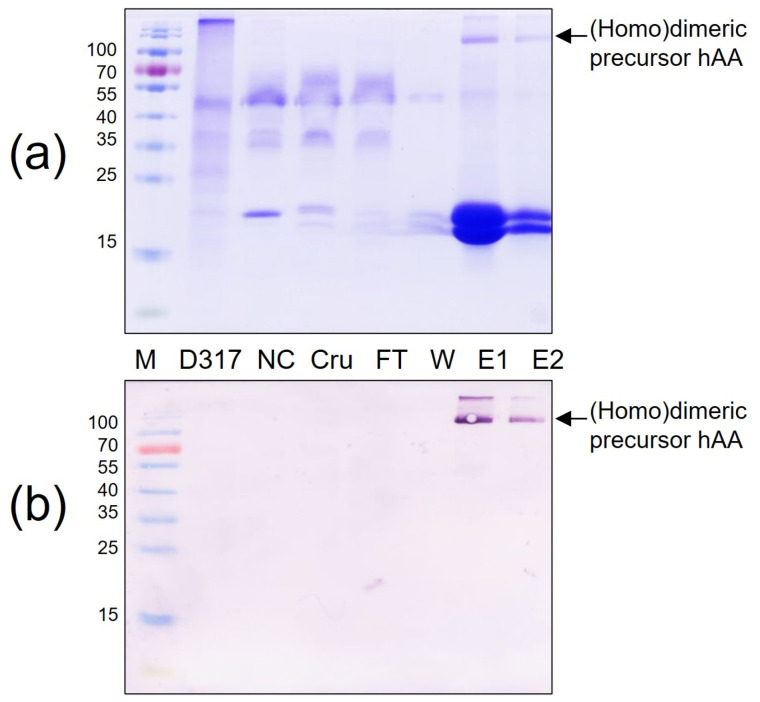
Purification of rhAA from transgenic rice suspension cell culture using a Ni^2+^—NTA agarose resin. Purified rhAA was visualized via SDS-PAGE (**a**) Western blot analysis (**b**). M, a pre-stained protein marker; PC, 100 ng of recombinant human AA; NC, culture medium from a non-transgenic cell line; Cru, culture medium after induction by sugar starvation (crude); FT, flow-through from Ni^2+^—NTA agarose resin affinity column; W, column flow-through washing buffer; E1 and E2, eluted fractions of rhAA.

The affinity chromatography on a Ni^2+^—NTA agarose column was utilized to obtain purified rhAA. One milliliter of elution buffer was added into the affinity chromatography column to release the column-bonded rhAA. The purity of His-tag fusion rhAA was examined by SDS-PAGE (Figure 6a) and Western blot analysis (Figure 6b). Results confirmed that rhAA could be purified from the supernatant of the suspension culture medium using Ni^2+^—NTA agarose resin collum.

### 3.6. Release Mature Form of Recombinant Human Activin A by Enterokinase Treatment

To obtain the mature (active) form of rhAA, an enterokinase cleavage site was inserted between the pro-region and mature region of rhAA. Hence, the mature form of rhAA was then obtained by treatment with enterokinase. Enterokinase is a highly specific serine protease and in vivo, enterokinase specifically hydrolyzes the lysine isoleucine bond of the activation peptide in precursor AA to yield active AA. The specific recognition sequence for enterokinase is X-Asp-Asp-Asp-Asp-Lys- ↓-Not Proline-X (X = any amino acid). 

**Figure 7:**
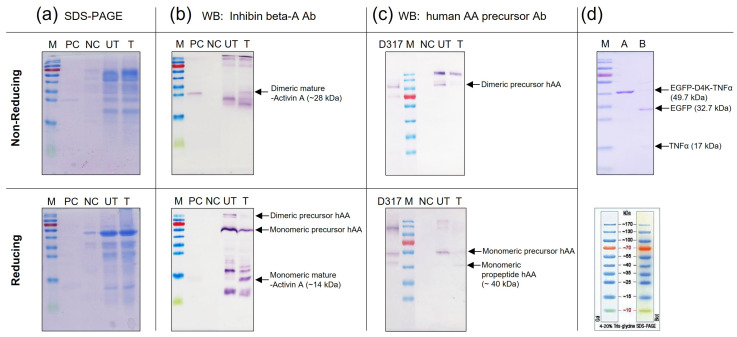
Detection of mature rhAA after enterokinase treatment via SDS-PAGE (**a**) and Western blot analysis (**b**,**c**). The digested activity of enterokinase on the control protein, EGFP-D4K-TNFα (**d**).

The concentrated samples produced in the induced rice culture medium were treated with recombinant enterokinase. The proteolyzed samples were examined by SDS-PAGE (Figure 7a) and Western blot analysis (Figure 7b,c). Under enterokinase treatment, the precursor form of rhAA was split into dimeric and monomeric mature forms with molecular weights of 28 kDa (under non-reducing conditions) and 14 kDa (under reducing conditions), which were detected by the inhibin beta-activin monoclonal antibody (Figure 7b). No signal bands of mature rhAA were detected on the lanes without enterokinase treatment. In addition, the monomeric of pro-activin A forms with molecular weights of 40 kDa (under reducing conditions) was detected using the human AA precursor Ab (only binding to the pro-region of rhAA), whereas it was not detected on un-treated samples (Figure 7c). The cleavage activity of enterokinase was also confirmed on the control protein, EGFP-D4K-TNFα. After enterokinase treatment, the fusion protein EGFP-D4K -TNFα was digested, showing two bands: one band of EGFP and one band of TNFα with molecular weights of 32.7 kDa and 17 kDa, respectively (Figure 7d). Taken together, the mature form of rhAA could be released from the precursor form using the specific recognition sequence for enterokinase, which fused between the pro-region and mature region of rhAA. 

## 4. Discussion

Activin A has been recombinantly expressed in various mammalian cell lines [1,19]. However, this approach may lead to issues, such as cell cytotoxicity [20] or the generation of high levels of uncleaved high molecular weight precursor forms of activin A [21]. Furthermore, the presence of native binding partners in these cell lines can result in an undesirable association of activin molecules with other cell surface proteins and multi-protein complexes [22]. Employing *Escherichia coli* as a host for expressing biologically active recombinant activin A is not viable, as prokaryotes lack the intracellular machinery necessary for the correct processing of the precursor protein into the mature, biologically active activin A molecule [23,24]. While activin A expressed in baculovirus-infected insect cells and is secreted into the culture medium and exhibits bioactivity, the yields are typically low, and the associated procedures are both costly and time consuming [22]. 

In the current study, mature recombinant human activin A (rhAA) was produced in a transgenic rice cell suspension culture. Transgenic rice cell calli transformed with a binary vector driven by the Ramy3D promoter system yielded rhAA at a level of 0.43 µg/mL at 13 days post-induction under sugar starvation (Figure 5e). Suspension cell culture stands as a widely employed method for the production of recombinant proteins, with upscaled systems integrated into bioreactors for commercial applications [25,26,27]. The Ramy3D promoter system has proven effective for producing various recombinant proteins in transgenic rice cell suspension cultures [28,29]. Notably, the successful production of recombinant human cytotoxic T-lymphocyte antigen 4-immunoglobulin (hCT-LA4Ig) has been achieved in rice suspension cells utilizing the Ramy3D promoter, reaching a maximum yield of 31.4 mg/L in a liquid medium [30]. The Ramy3D promoter system enables the efficient production of various recombinant proteins in suspension culture, with substantial secretion into the culture medium. For example, using this approach, various recombinant proteins such as acid alpha-glucosidase (GAA) at 37 mg/L [31], human serum albumin (HSA) at 76.4 mg/L [32], and CCP monoclonal antibody (CCP mAb) at 22 mg/L [17] were produced in a transgenic rice cell suspension culture. In another study, Huang et al. [33] reported that using the RAmy3D promoter system in a rice suspension cell culture, recombinant human octamer-binding transcription factor 4 (Oct4) reached the highest yield of approximately 0.41% of total cellular soluble proteins after one day of induction. These exploits showcased the potential of transforming plants into bio-factories capable of large-scale production of recombinant proteins.

The mature form of activin A is a biologically active dimeric C-terminal cleavage product with a molecular weight of 28 kDa, requiring full processing to become functional. The β-subunit precursor consists of a 290 amino acid pro-region and a 116 amino acid C-terminus [1]. The lengthy N-terminal pro-peptide region is subsequently removed, resulting in the formation of a mature dimeric activin A, characterized by nine intra- and inter-chain disulfide bonds, which are crucial for its bioactivity [34]. A biosynthetic study indicates that the N-terminal pro-peptide of activin A plays a vital role in correct disulfide bond formation and the secretion of an active dimer. Typically, two precursor chains are di-sulfide-linked to form covalent dimers through conserved cysteine residues in the mature domains. During the secretory pathway, furin-like pro-protein convertases process the precursors at a polybasic RRRRR motif [21]. This motif is putatively cleaved by subtilisin-like pro-protein convertases (SPCs), like furin, releasing the mature signaling domain from the covalent linkage to the pro-domain [35]. Mutations in this furin site underscore the necessity of cleavage for growth factor activation. Human activin A was expressed in *Pichia pastoris*, allowing its secretion into the culture medium and purification as the mature homodimer [3]. The expression vector was engineered to encode the monomeric precursor protein with an N-terminal FLAG affinity tag (DYKDDDDK) and a cleavage site (EKR) for Kex2p protease (enterokinase). Pro-domains are essential for the biosynthesis, stabilization, transportation, and signaling of growth factors, as demonstrated by their importance in the assembly and secretion of dimeric activin A [36]. Disease-causing mutations have been identified in various pro-domains, emphasizing their crucial role in the biology of these growth factors [37]. The complex disulfide structure and the involvement of the pro-peptide in native activin formation pose a significant challenge for refolding, making this protein an intriguing candidate for testing the reverse screening approach [23]. Previous studies have demonstrated the production of human TGFβ1 in *Nicotiana benthamiana*, the mature form with biological activity obtained after enterokinase treatment [38]. Based on the specific cleavage property of enterokinase, the fusion protein is released after treatment with this enzyme [39,40]. In this study, to produce bioactive activin A, a cleavage site of enterokinase (D4K) was introduced between the pro-region and mature region of rhAA. Following enterokinase treatment, mature activin A was released (Figure 7b,c).

## 5. Conclusions

In this study, the expression vectors harbored the precursor form of rhAA containing cleavage sites of enterokinase were constructed to obtain the mature form of rhAA protein. rhAA was expressed and secreted onto a suspension culture medium. The highest expression level in the rice suspension culture reached 0.43 ug/mL at 13 days of induction. Purified recombinant hAA was obtained through affinity chromatography Ni^2+^—NTA. Initial results show that the mature (active) form of rhAA can be released from precursor rhAA by inserting a recognition cleavage site (D4K sequence) between the pro-region and mature region of rhAA. These results suggest that a transgenic rice cell suspension culture can be employed to produce rhAA. Additionally, inserting the recognition cleavage site of D4K can be applied to other fusion proteins that need proteolytically processing with enterokinase to obtain the desired protein thereafter. Overall, this study demonstrates successful expression, purification, and maturation of rhAA protein using a rice calli suspension culture. However, further studies are necessary to analyze the bioactivity of mature rhAA, which is crucial for its potential biomedical applications.

## Figures and Tables

**Table 1:** Primer sequences for cloning recombinant human activin A.

Primer No ^z^	Primer Name	Sequence (5′→3′)
1	ActA_R	CTCGAGGGTCTCAAAGCGGTAC
2	ActA_F	AACTCGGGCCAAGCCGGCCTCGAATGCGACGGC
3	ActA1_F	GGTTAATTAAGGTCTCAAGGTTC
4	ActA1_R	ATGAGGGTGGTCTTCAGACTG
5	ActA2_F	TCTGAAGACCACCCTCAT**GACGATGATGACAAG**GGCCTCGAATGC GACGGC

^z^ To amplify, the pro-region of AA used primer sets 3 and 4; the mature region, including the enterokinase cleavage site (D4K), used primer sets 1 and 5; the full gene used primer sets 3 and 1 by overlap PCR. Underline with bold sequence indicates the sequence of the enterokinase cleavage site.

## Data Availability

Data are contained within the article.
